# Molecular analysis of the effects of steroid hormones on mouse meiotic prophase I progression

**DOI:** 10.1186/s12958-019-0548-x

**Published:** 2019-12-02

**Authors:** Deion M. Burks, Margaret R. McCoy, Sudipta Dutta, Connie J. Mark-Kappeler, Patricia B. Hoyer, Melissa E. Pepling

**Affiliations:** 1 Department of Biology, Syracuse University, 107 College Place, Syracuse, NY 13244 USA; 2 Present address: University of Nebraska Medical Center, Omaha, NE USA; 3 Department of Physiology, College of Medicine, The University of Arizona, Tucson, AZ USA; 4 Present address: PRA Health Sciences Lenexa, Lenexa, KS USA

**Keywords:** Fetal oocyte development, Steroid hormones, Meiotic prophase, Diplotene arrest, Primordial follicle formation

## Abstract

**Background:**

Infertility is linked to depletion of the primordial follicle pool consisting of individual oocytes arrested at the diplotene stage of meiotic prophase I surrounded by granulosa cells. Primordial germ cells, the oocyte precursors, begin to differentiate during embryonic development. These cells migrate to the genital ridge and begin mitotic divisions, remaining connected, through incomplete cytokinesis, in clusters of synchronously dividing oogonia known as germ cell cysts. Subsequently, they enter meiosis, become oocytes and progress through prophase I to the diplotene stage. The cysts break apart, allowing individual oocytes to be surrounded by a layer of granulosa cells, forming primordial follicles each containing a diplotene arrested oocyte. A large number of oocytes are lost coincident with cyst breakdown, and may be important for quality control of primordial follicle formation. Exposure of developing ovaries to exogenous hormones can disrupt cyst breakdown and follicle formation, but it is unclear if hormones affect progression of oocytes through prophase I of meiosis.

**Methods:**

Fetal ovaries were treated in organ culture with estradiol, progesterone, or both hormones, labeled for MSY2 or Synaptonemal complex protein 3 (SYCP3) using whole mount immunocytochemistry and examined by confocal microscopy. Meiotic prophase I progression was also followed using the meiotic surface spread technique.

**Results:**

MSY2 expression in oocytes was reduced by progesterone but not estradiol or the hormone combination. However, while MSY2 expression was upregulated during development it was not a precise marker for the diplotene stage. We also followed meiotic prophase I progression using antibodies against SYCP3 using two different methods, and found that the percent of oocytes at the pachytene stage peaked at postnatal day 1. Finally, estradiol and progesterone treatment together but not either alone in organ culture increased the percent of oocytes at the pachytene stage.

**Conclusions:**

We set out to examine the effects of hormones on prophase I progression and found that while MSY2 expression was reduced by progesterone, MSY2 was not a precise diplotene stage marker. Using antibodies against SYCP3 to identify pachytene stage oocytes we found that progesterone and estradiol together delayed progression of oocytes through prophase I.

## Background

In mammals, the primordial follicle pool present at birth represents the total germ cell population available to a female during her entire reproductive life. The differentiation of primordial germ cells into functional oocytes contained in primordial follicles is poorly understood. In the mouse embryo, primordial germ cells migrate to the female genital ridge and are then referred to oogonia once they colonize the ovary [18]. Oogonia develop in connected clusters known as cysts until 13.5 days post coitum (dpc) in the mouse and then become oocytes as they begin to enter meiosis [19]. Meiotic entry occurs in a wave from the anterior to the posterior side of the ovary [2, 15]. The oocytes proceed through prophase I of meiosis progressing through a series of sub-phases starting with pre-meiotic interphase and then moving through leptotene, zygotene, pachytene, and eventually arresting at the diplotene stage [1]. The extended diplotene arrest often lasting years (until ovulation) is sometimes referred to as dictyate [5]. The germ cells enter diplotene arrest beginning at 17.5 dpc, which corresponds with the start of cyst breakdown [1, 8, 21]. As more germ cells arrive at diplotene and cysts begin to break down to form primordial follicles, some oocytes experience programmed cell death [20]. In late fetal and early neonatal development, cysts break apart into individual oocytes and are surrounded by somatic pre-granulosa cells [13, 20]. This results in primordial follicles each consisting of a diplotene arrested oocyte enclosed by several granulosa cells.

Mutations that disrupt meiotic prophase I progression in females affect fertility. For example, in females mutant for genes involved in recombination and repair, the germ cells do not progress beyond the pachytene stage of prophase I and eventually die resulting in infertility [18]. Also, inhibition of Synaptonemal Complex Protein 1 mRNA (*Sycp1*), found in synaptonemal complexes which are protein structures that hold the homologous chromosome pairs together at the pachytene stage, caused premature arrival at the diplotene stage and premature primordial follicle formation suggesting a link between cell cycle stage and primordial follicle formation [17]. However, in *Stra8* mutants meiotic entry is blocked but primordial follicles still form implying that meiosis and follicle formation are independent [7]. We found a small subset of primordial follicles with oocytes at prediplotene stages supporting the idea that oocytes do not have to reach the diplotene stage before follicles form [8].

Previous work from our lab demonstrated that estrogen or progesterone can reduce cyst breakdown and primordial follicle formation and together have an additive effect [3]. There is also some evidence that steroid hormones can affect progression through meiotic prophase I. For example, in cows, high levels of estradiol (E_2_) and progesterone (P_4_) were associated with a delay in reaching the diplotene stage [26]. Supporting this, treatment of mouse embryos with the estrogenic compound, bisphenol A (BPA) caused defects in meiosis suggesting that E_2_ signaling could be involved in regulating meiotic progression [23]. Estrogen receptor 2 (*Esr2*) mutants had meiotic defects similar to BPA treated animals suggesting that BPA acts as an ESR2 antagonist. Work from our lab found that in fetal organ culture progesterone but not estradiol delayed transit though meiotic prophase I [8].

One of the most common techniques used for meiotic staging is the histological method of hematoxylin and eosin (H&E) staining that requires embedding in paraffin, sectioning, staining and then analysis of ovary sections for meiotic stage. This method is tedious, time consuming and results in a loss of three dimensional structural information. A more recently used molecular technique is the surface spread assay involving lysis of the ovary to open the cells allowing labeling of chromosomes with a fluorescent marker. While this method greatly improves the ability to identify oocytes in stages of meiotic prophase I, the ovary is completely disaggregated resulting in the loss of any structural information. One reported molecular diplotene arrest marker is MSY2, an RNA binding protein expressed in germ cells [9]. MSY2 is believed to be involved in regulating mRNA stability in growing oocytes and when the gene is deleted, females become sterile [24]. Several groups have used MSY2 protein expression as an indicator that an oocyte has reached the diplotene stage of meiotic prophase I [17, 22]. Another protein used as a marker for meiotic prophase I staging is SYCP3. As the oocytes reach pachytene, the synaptonemal complex forms holding homologous chromosomes together and SYCP3 localizes in between the chromosomes. At diplotene, homologous chromosomes begin to separate remaining attached only at points of crossing over and SYCP3 becomes diffuse except for a few sites of strong staining [4, 6].

The objective of the work presented here was to test effects of steroid hormones, estrogen and progesterone on meiotic prophase I progression using available molecular tools.

## Materials and methods

### Animals

CD-1 mice used for RNA studies were obtained from Charles River Laboratories and C57BL/6 mice used for all other studies were obtained from Jackson Laboratories. Mice were housed and bred at a controlled photoperiod (14 h light, 10 h dark), temperature (21–22 °C), and humidity with food and water available ad libitum. Females were mated with males of the same strain and checked daily for vaginal plugs. Noon on the day of vaginal plug detection was designated as 0.5 dpc. Birth usually occurred at 19.5 dpc and was designated as postnatal day (PND) 1. Pregnant mice were euthanized by CO_2_ asphyxiation for fetal ovary collection. For neonatal ovary collection, pups were euthanized by decapitation on the appropriate day. All animal protocols were approved by the Syracuse University Institutional Animal Care and Use Committee.

### Study design

Mouse ovary organ culture was used to investigate the effects of hormones on oocyte meiotic prophase I progression. Ovaries were harvested at 17.5 dpc and cultured for 5 days in DMSO, E_2_, P_4_ or both hormones at 10^− 6^ M. Ovaries were collected and labeled with antibodies against MSY2 and TRA98 or SYCP3 and VASA using immunocytochemistry. *Msy2* mRNA expression during fetal and neonatal oocyte development was examined using RT-PCR. The expression of MSY2 and SYCP3 protein was followed during oocyte development using whole mount immunocytochemistry. SYCP3 protein was also followed over time using the meiotic surface spread technique.

### RNA isolation

Fetal (13.5 dpc-18.5 dpc) and neonatal (PND1-PND5) ovaries were dissected in PBS, placed in RNA*later*, flash frozen in liquid nitrogen and stored at − 80 °C. Total RNA was isolated using Qiagen’s RNeasy Mini kit following the manufacturers instructions (*n* = 3; 50 or 100 ovaries per pool for neonatal and fetal ovaries respectively). Ovaries were briefly lysed and homogenized using a motor pestle on ice and the mixture was then applied to a QIAshredder column. The ovarian tissue sample in the QIAshredder column was then centrifuged at 11,000 g for 2 min. To isolate the RNA, the resulting flow-through was applied to an RNeasy mini column which allowed the RNA to bind to the filter cartridge. RNA was eluted by washing from the filter and was concentrated using an RNeasy MinElute kit. The RNA which was isolated was briefly applied to an RNeasy MinElute spin column and after washing, RNA was eluted using 14 μl of RNase-free water. The RNA concentration in the elutant was determined using an ND-1000 Spectrophotometer (λ = 260/280 nm; Nanodrop Technologies, Inc., Wilmington, DE).

### First strand cDNA synthesis and real-time polymerase chain reaction (PCR)

Total RNA (0.5 μg) was reverse transcribed into cDNA using the Superscript III One-Step RT-PCR System. The cDNA was diluted in RNase-free water (1:25). 2 μl of diluted cDNA was amplified on a Rotor-Gene 3000 using Quantitect™ SYBR Green PCR kit and custom designed primers for *Msy2* (forward primer: 5′ CCC TGG CAA CCA GGC GAC GG 3′; reverse primer: 5′ TGA CTG TGC CCA GGA CTT GGA TTG 3′; NCBI Genbank accession number NM_016875), and β-actin (forward primer: 5′ AGT GTG ACG TTG ACA TCC GTA 3′; reverse primer: 5′ GCC AGA GCA GTA ATC TAA TTA T 3′; NCBI Genbank accession number NM_007393). The cycling program consisted of a 15 min hold at 95 °C and 45 cycles of: denaturing at 95 °C for 15 s, annealing at 58 °C for 15 s, and extension at 72 °C for 20 s at which point data were acquired. Determination of product melt conditions was done using a temperature gradient from 72 °C to 99 °C with a 1 °C increase at each step. β-actin expression remained constant across all ages and therefore each sample was normalized to β-actin before quantification.

### Immunocytochemistry

Once ovaries were harvested, they were fixed with 5.3% EM grade formaldehyde in PBS overnight at 4 °C and immunostained as previously described [16]. Briefly, ovaries went through a series of washes at room temperature in 0.1% Triton X-100 in 1X PBS (PT) and then PT + 5% bovine serum albumin (BSA). Following washes, ovaries were incubated overnight with primary antibodies diluted in PT + 5% BSA at 4 °C (see Table 1 for antibodies and dilutions). After overnight incubation in primary antibodies, ovaries were washed in PT + 1% BSA treated with RNase A and labeled with propidium iodide or TOTO3. Ovaries were then incubated with pre-absorbed secondary antibodies (see Table 2 for secondary antibodies) at a dilution of 1:200 overnight at 4 °C. Negative controls using only secondary antibodies were previously tested in the lab for all antibodies used. Ovaries were washed in PT + 1% BSA, rinsed in PBS, placed in Vectashield, mounted and observed by confocal microscopy on a Zeiss LSM 710 confocal microscope.

**Table 1:** Primary Antibodies and Dilutions Used

Primary Antibody	Company	Catalog #	Species Made In	Dilution
MSY2 (C-15)	Santa Cruz Biotechnology	sc-21,316 Lot #: C0615	Goat	1:100
SYCP3 (D-1)	Santa Cruz Biotechnology	sc-74,569 Lot #: J1314	Mouse	1:200 (whole mount) 1:50 (surface spreads)
TRA98	B-Bridge	73–003 Lot #: 141113A	Rat	1:500
VASA	Abcam	ab-13,840 Lot #: GR172688–1	Rabbit	1:250

**Table 2:** Secondary Antibodies and Dilutions Used

Secondary Antibody	Company	Catalog #	Species Made In	Dilution
Alexa Fluor 488 Donkey Anti-Goat	Molecular Probes/Invitrogen	A11055	Donkey	1:200
Alexa Fluor 488 Donkey Anti-Rat	Molecular Probes/Invitrogen	A21208	Donkey	1:200
Alexa Fluor 488 Goat Anti-Mouse	Molecular Probes/Invitrogen	A11029	Goat	1:200
Alexa Fluor 488 Goat Anti-Rabbit	Molecular Probes/Invitrogen	A11008	Goat	1:200
Alexa Fluor 568 Donkey Anti-Goat	Molecular Probes/Invitrogen	A11057	Donkey	1:200
Alexa Fluor 568 Goat Anti-Mouse	Molecular Probes/Invitrogen	A11004	Goat	1:200

### Meiotic surface spreads

Ovaries were harvested, incubated in Hypotonic Extraction Buffer (30 mM Tris, 50 mM sucrose, 17 mM trisodium citrate dihydrate, 5 mM EDTA, 0.5 mM DTT, and 0.5 mM phenylmethylsulphonyl fluoride (PMSF), pH 8.2) and then teased apart in 100 mM sucrose. The cell suspension was dried down and fixed in 1% paraformaldehyde. Slides were incubated overnight in a humidity chamber at 37 °C. Slides were then air dried, washed in 0.4% PhotoFlo, air dried again and stored at − 20 °C until staining. Slides were washed in PBS, blocked with 2.5% goat serum and stained with antibodies against SYCP3 (see Table 1) diluted in 2.5% goat serum in a humidity chamber overnight at 4 °C. Subsequently, slides were washed with 0.1% Tween in PBS and then incubated Alexa Fluor 488 Goat Anti-Mouse secondary antibodies (see Table 2) diluted in 2.5% goat serum for 1 hour. Slides were washed with 0.1% Tween in PBS, mounted in a 1:1 solution of Vectashield and 2 μg/ml DAPI and stored at − 20 °C.

### In vitro ovary organ culture

Ovaries dissected at 17.5 dpc were placed in culture. Ovaries were cultured in 4-well culture plates in drops of media on 0.4 μM floating filters (Millicell- CM; Millipore Corp., Bedford, MA) in 0.4 ml DMEM-Ham’s F-12 media supplemented with penicillin-streptomycin, 5X ITS-X (Life Technologies, Inc., Grand Island, NY), 0.1% BSA, 0.1% albumax, and 0.05 mg/ml L-ascorbic acid. E_2_ and P_4_ (Sigma Chemical Co., St. Louis, MO) were dissolved in dimethylsulfoxide (DMSO) at a concentration of 0.1 M and then added to culture media to achieve the desired final concentration. DMSO was added to media at the same percentage as a vehicle control. Ovaries were exposed daily to DMSO, E_2_, P_4_ or both hormones at 10^− 6^ M (*n* = 5 ovaries per treatment group). Ovaries were divided randomly among the treatment groups. Ovaries were fixed in formaldehyde and immunostained as described above.

### Statistical analysis

Data are represented as mean ± SEM of nontransformed data. Statistical analyses using transformed data were performed using GraphPad Prism version 6 (GraphPad Software, San Diego, CA). Statistical differences (*P* < 0.05) among the means were evaluated using one-way ANOVA followed by Newman-Keuls multiple comparisons test. Effects of E_2_ and P_4_ on MSY2 and SYCP3 expression were analyzed using one-way ANOVA followed by Dunnett’s multiple comparisons test. Statistical analyses of real time PCR data were performed using Statview 5.0.1 (SAS Institute Inc., Cary, NC). Differences between fold increases in mRNA levels over various time points were evaluated by one-way ANOVA followed by Bonferroni-Dunn’s post hoc test (*P* < 0.0005).

## Results

### Exposure to steroid hormones alters Msy2 expression

**Fig. 1:**
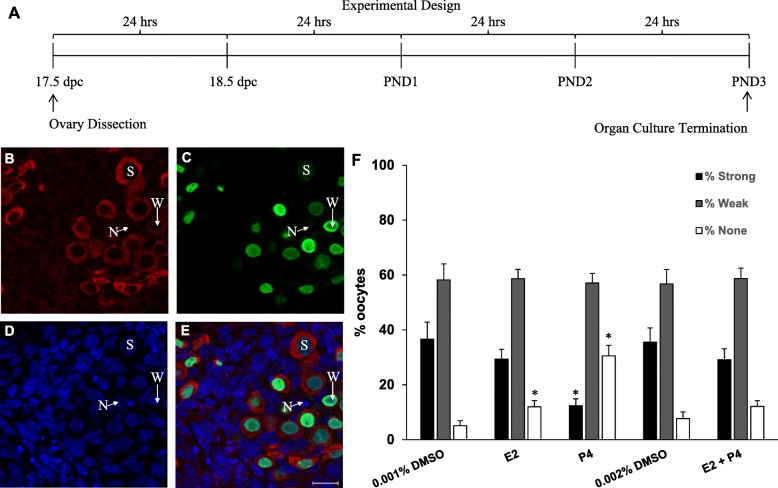
Effects of exogenous estradiol and progesterone on MSY2 expression. **a** Experimental design of organ culture experiment. **b**-**e** Representative confocal section labeled with **b** MSY2 (red), **c** TRA98, oocyte marker (green), **d** TOTO3, nuclear marker (blue) and **e** overlay. Oocytes marked as strong (S), weak (W), or no (N) expression. Scale bar = 20 μm. **f** Graph showing the percentage of oocytes expressing MSY2 strongly, weakly or not at all (+/−SEM). Asterisk indicates significant difference from control (*P* < 0.05; *n* = 8 ovaries per treatment group) as determined by a two-tailed T-test

Our lab previously showed that exogenous exposure of developing ovaries to estradiol or progesterone reduced cyst breakdown and follicle formation and together had an additive effect [3]. Here, we examined effects of exogenous hormone exposure on meiotic prophase I progression of oocytes from fetal ovaries by treatment with estradiol and/or progesterone. 17.5 dpc ovaries were harvested and grown in vitro using an organ culture system. Ovaries were grown for 5 days in DMSO, 10^− 6^ M estradiol, 10^− 6^ M progesterone, or 10^− 6^ M estradiol + progesterone until they reached the equivalent of PND3 (Fig. 1a). An antibody against MSY2, a reported marker of diplotene arrest was used to follow meiotic progression. Ovaries were fixed and labeled with MSY2 and the oocyte marker, TRA98 using immunocytochemistry and then analyzed by confocal microscopy (Fig. 1b-e). Oocytes were marked as either expressing MSY2 strongly, weakly, or not at all in order to analyze the effects of hormone exposure on meiotic progression. Progesterone significantly lowered the percent of oocytes strongly expressing MSY2 and significantly increased the percent of oocytes with no MSY2 expression (Fig. 1f). Estradiol alone as well as the combination of estradiol and progesterone did not have a significant impact on MSY2 expression.

### MSY2 expression correlates with diplotene arrest but is not a diplotene stage marker

**Fig. 2:**
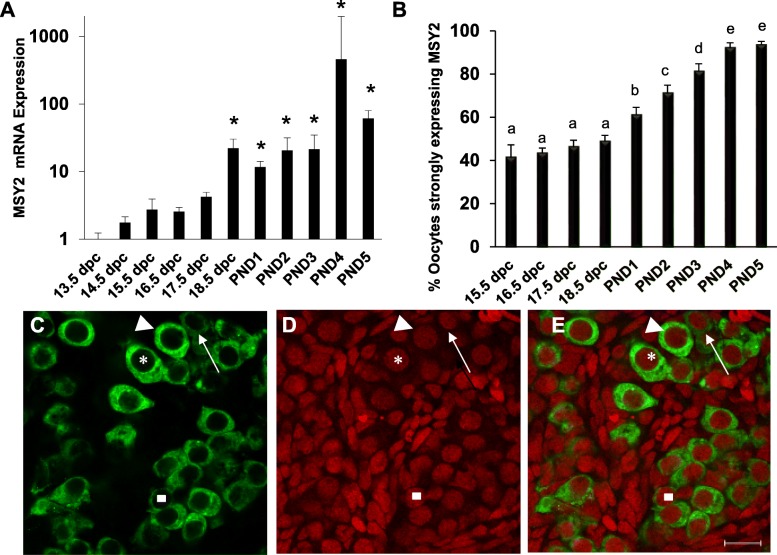
MSY2 expression increases over time in developing ovaries and is asymmetric. **a** Relative mRNA levels (+/−SEM) for *Msy2* as determined by qPCR in perinatal mouse ovaries from 13.5 dpc to PND5. The levels of *Msy2* mRNA are normalized to the levels of mRNA for a housekeeping gene, β-actin in the same sample. The normalized values are expressed relative to the mRNA levels at 13.5 dpc with 13.5 dpc set at 1. Asterisks indicate a significant difference (*P* < 0.0005) as determined by one-way ANOVA with Bonferroni-Dunn’s post hoc test (*n* = 3). **b** Percent of oocytes with strong MSY2 protein labeling in ovaries (+/−SEM) from 15.5 dpc through PND5. Different letters indicate a significant difference between groups (*P* < 0.05; *n* = 8 ovaries per developmental stage) as determined by a one-way ANOVA followed by Newman-Keuls multiple comparisons test. **c**-**e** Asymmetric expression of MSY2 protein in wildtype ovaries. Image shown is a confocal microscope section from a PND1 wildtype ovary. **c** MSY2 expression labeled in green. **d** Nuclei of all cells labeled with propidium iodide in red. **e** Overlay of both MSY2 (green) and propidium iodide (red) channels. Asterisk indicates an oocyte strongly expressing MSY2. Square indicates an oocyte with weak MSY2 expression. The arrow and arrowhead are showing an example of asymmetric expression of MSY2 within the same cyst. The arrow indicates an oocyte with weak MSY2 expression within the cyst and the arrowhead indicates an oocyte with strong MSY2 expression within that same cyst. Scale bar = 20 μm

MSY2 expression has previously been used to indicate arrest at the diplotene stage of meiotic prophase I [17, 22]. To confirm that MSY2 is a diplotene arrest marker we examined MSY2 mRNA and protein expression during fetal and neonatal oocyte development. First, we measured the levels of *Msy2* mRNA by qPCR in ovaries from 13.5 dpc to PND 5 (Fig. 2a). A slight increase of *Msy2* mRNA is observed at 17.5 dpc with a statistically significant increase at 18.5 dpc correlating with the increase in diplotene oocytes observed by our lab and others starting at 17.5 dpc [1, 8]. We also examined MSY2 protein expression using whole mount immunostaining in ovaries from 15.5 dpc through PND 5. At each timepoint, oocytes were counted and marked as either strongly expressing or weakly expressing MSY2 (Fig. 2c-e). Strong expression of MSY2 within an oocyte was taken to mean that the cell had reached and arrested at the diplotene stage of meiotic prophase I. Approximately 40% of the oocytes strongly expressed MSY2 at 15.5 dpc and this increased over time to PND5 when almost all oocytes (~ 94%) were strongly expressing MSY2 (Fig. 2b). The increase in oocytes strongly expressing MSY2 correlates with the expected increase in oocytes arriving at and arresting at the diplotene stage of meiotic prophase I. However, we were surprised that 40% of the oocytes were already expressing MSY2 at high levels even though it is known that oocytes don’t start to arrive at diplotene until 17.5 dpc thus we conclude that while MSY2 expression correlates with arrival at the diplotene stage it is not a marker for diplotene arrest per se.

### SYCP3 can be used to follow meiotic progression in surface spreads and in whole mount immunocytochemistry

**Fig. 3:**
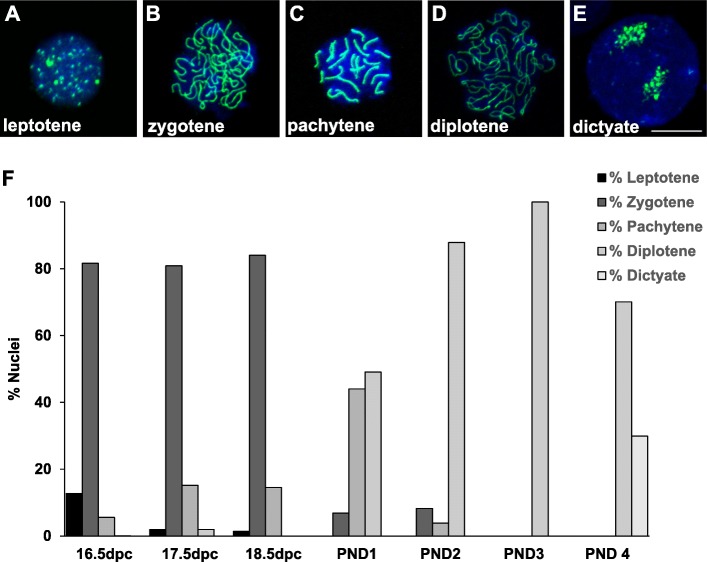
Analysis of meiotic prophase I progression using surface spreads. **a-e** Examples of nuclei at each substage of prophase one labeled with SYCP3 (green) and DAPI (blue). **a** Nucleus in the leptotene substage showing chromosomes beginning to condense. **b** Nucleus in the zygotene substage showing homologous chromosomes beginning to come together and the synaptonemal complex starting to form. **c** A pachytene nucleus showing completion of synapsis. **d** A diplotene arrested nucleus showing chromosomes that have begun to separate but remain attached at sites of crossing over. **e** A late-diplotene/dictyate nucleus, with chromosomes condensed and at opposite poles. Scale bar = 20 μm. **f** Percent of oocyte nuclei in each stage of prophase one from 17.5 dpc to PND4 determined using the meiotic surface spread assay with SYCP3 staining. Approximately 200 nuclei were counted for each age of development

We investigated alternative methods to follow meiotic progression including the surface spread technique. This method allows a more precise identification of meiotic prophase I substage but involves the disassociation of the tissue resulting in the loss of any cellular structure. Nuclei prepared using the surface spread technique are then labeled with an antibody against SYCP3 which labels the synaptonemal complex formed between homologous chromosomes at the pachytene stage. This labeling can be used to stage nuclei and oocytes labeled with SYCP3 using this technique at each stage of meiotic prophase I are shown in Fig. 3a-e. We used this technique to determine the percent of oocytes at each stage from 16.5 dpc to PND4 (Fig. 3f). We found the peak percentage of oocytes in the pachytene stage was 44% at PND1.

**Fig. 4:**
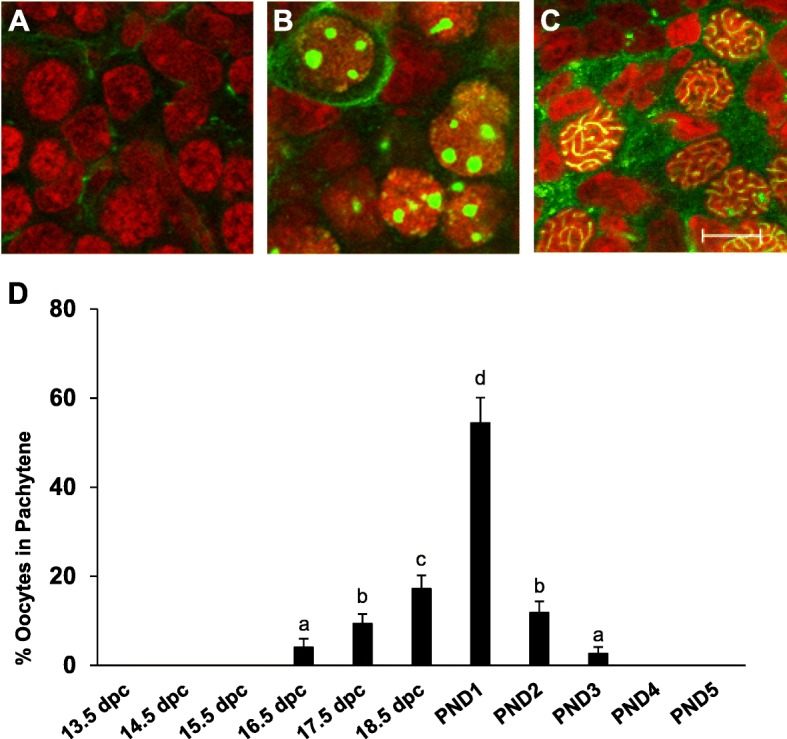
The percentage of pachytene oocytes peaks at PND1 as determined by SYCP3 labeling. **a-c** Representative confocal microscope sections labeled for SYCP3 (green) and nuclear marker propidium iodide (red) at **a** 13.5 dpc showing unlabeled mitotic nuclei, **b** 15.5 dpc showing pre-pachytene meiotic nuclei and **c** PND1 showing pachytene meiotic nuclei. Scale bar = 10 μm. **d** Percent of oocytes in the pachytene stage of development in ovaries from 13.5 dpc through PND5 determined by whole mount SYCP3 expression (+/−SEM). Different letters indicate a significant difference between groups (*P* < 0.05; *n* = 8 ovaries per developmental stage) as determined by a one-way ANOVA followed by Newman-Keuls multiple comparisons test

We also examined the expression of SYCP3 in whole mount immunohistochemistry which is upregulated as the germ cells enter meiosis and at the pachytene stage has a very striking localization as condensed “strings” between homologous chromosomes (Fig. 4a-c). SYCP3 expression was analyzed within the female germ cells from 13.5 dpc through PND 5 and the percent of pachytene stage oocytes was determined (Fig. 4d). Oocytes in the pachytene stage were not observed until 16.5 dpc. After 16.5 dpc, the number of pachytene labeled oocytes increased until PND1 where a peak of approximately 55% was observed. Starting at PND2, the number of oocytes at the pachytene stage significantly decreased and no cells were observed to be in pachytene after PND3.

### Exposure to steroid hormones delays progression through prophase I

**Fig. 5:**
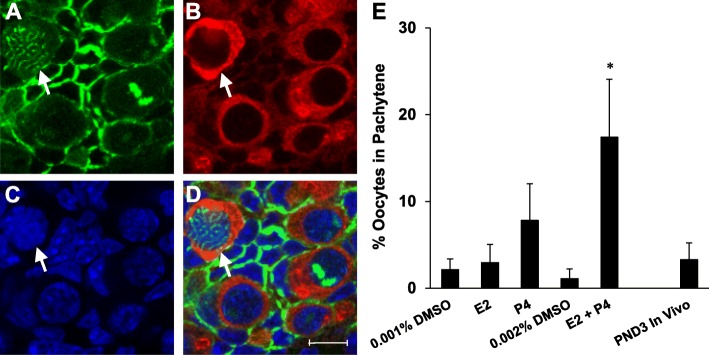
Effects of estradiol and progesterone on progression through meiotic prophase I. **a-d** Representative confocal section labeled with **a** SYCP3 (green) showing oocyte with pachytene expression pattern (arrow), **b** VASA, oocyte marker (red), **c** TOTO3, nuclear marker (blue) and **d** overlay. Scale bar = 10 μm. **e** Graph showing the percentage of oocytes in the pachytene stage as determined by SYCP3 expression pattern (+/−SEM). Asterisk indicates significant difference from control (*P* < 0.05; *n* = 8 ovaries per treatment group) as determined by a two-tailed T-test

17.5 dpc ovaries were again harvested and cultured for 5 days with DMSO vehicle, 10^− 6^ M estradiol, 10^− 6^ M progesterone, and 10^− 6^ M estradiol + progesterone until they reached PND3 similar to Fig. 1a. This time, ovaries were labeled for SYCP3 and the germ cell marker, VASA using whole mount immunocytochemistry and then analyzed by confocal microscopy (Fig. 5a-d). The percent of pachytene oocytes was determined in order to analyze the effects of hormone exposure on meiotic progression. Ovaries treated with estradiol and progesterone together had a significant increase in the percent of oocytes in the pachytene stage suggesting that progression through meiotic prophase I was delayed (Fig. 5e).

## Discussion

Here, we show that MSY2 expression in oocytes is reduced by progesterone but not estradiol alone or estradiol and progesterone in combination using ovary organ culture. In addition, while MSY2 upregulation correlates with arrival at the diplotene stage, is a not a diplotene stage marker as some pre-diplotene oocytes highly express MSY2. SYCP3 can be used to follow progress of oocytes through meiotic prophase I using both surface spreads and whole mount immunostaining with the pachytene stage especially prominent. Finally, estradiol and progesterone together but not either hormone individually delay prophase I progression as determined by the expression pattern of SYCP3 in whole mount immunostaining.

Our data suggest that MSY2 protein levels increase as oocytes approach the diplotene phase of meiotic prophase I but is not a diplotene stage marker per se. As shown in Fig. 2b, MSY2 is strongly expressed in oocytes even at 15.5 dpc before any oocytes have reached the diplotene stage. MSY2 is a conserved RNA binding protein specifically expressed in germ cells and required for fertility [9, 24]. In male germ cells it is important post-meiotically during spermiogenesis [25]. In oocytes, MSY2 protein regulates mRNA stability as oocyte increases in size [14]. *Msy2* mutant oocytes have many abnormalities such as aberrant spindle formation and chromosome congression during meiosis II, however, there is no evidence for a role of MSY2 in meiotic prophase I. Likely, the expression of MSY2 is upregulated during meiotic prophase I in preparation for future oocyte growth and later meiotic functions.

Previous research has shown that estrogen and progesterone have negative effects on the development process of female germ cells. Progesterone and estradiol, the phytoestrogen genistein, as well as synthetic estrogens all disrupt cyst breakdown and follicle formation [3, 11, 12] thereby leading to a potential decrease in viable egg cells later in life. In some instances, the number of oocytes present is also affected, but not as consistently. Here, ovaries were treated with estradiol alone, progesterone alone, or both estradiol and progesterone and effects on meiotic progression examined using SYCP3 expression in whole mount immunostaining. The number of oocytes found in the pachytene stage significantly increased only in the estradiol and progesterone treatment group indicating that treatment with both hormones delayed meiotic progression. Previous research has shown that when pregnant female mice were exposed to Bisphenol A (BPA), an estrogenic chemical, meiotic progression was disrupted by way of a disturbance of the synapsis and recombination of chromosome homologs [23]. Another study showed that progesterone acts through the progesterone receptor membrane component 1 (PGRMC1) to significantly delay or completely disrupt meiotic progression and therefore disrupt primordial follicle assembly [10]. The results of our organ culture agreed with these findings.

Our previous work investigating prophase I progression using standard histology demonstrated that progesterone but not estradiol or the combination of estradiol and progesterone delayed meiotic progression [8]. However, in the work presented here only the combination of estradiol and progesterone significantly affected progression through meiosis. One difference between the two studies is the mouse strain used. The CD1 outbred strain was used in our earlier study while here we used the B6 inbred strain. Interestingly, we also found that only progesterone alone reduced MSY2 expression.

We used two different molecular techniques to follow prophase I progression during perinatal oocyte development. The surface spread technique combined with a synaptonemal complex marker such as SYCP3 allows precise identification of prophase I substages. However, all structural information is lost with this method. The second technique, also using SYCP3 is whole mount immunostaining which preserves three dimensional structural information but does not allow precise identification of prophase substages though the pachytene stage is easy to identify. Using both techniques we found the largest percentage of pachytene oocytes at PND1 (~ 45% in surface spreads and ~ 55% in immunostaining). Thus, both techniques were able to be used to provide information regarding meiotic progression.

## Conclusions

The ultimate outcome of perinatal oocyte development is the formation the ovarian reserve consisting of a pool of primordial follicles with each follicle containing a diplotene arrested oocyte. Here we demonstrated the impact of steroid hormone signaling on meiotic prophase I progression. Future work investigating additional mechanisms regulating progression through meiotic prophase I will be important to understand the production of a robust ovarian reserve.

## Data Availability

The datasets analyzed during the current study are available from the corresponding author on reasonable request.
